# Application of improved YOLOv7-based sugarcane stem node recognition algorithm in complex environments

**DOI:** 10.3389/fpls.2023.1230517

**Published:** 2023-08-23

**Authors:** Chunming Wen, Huanyu Guo, Jianheng Li, Bingxu Hou, Youzong Huang, Kaihua Li, Hongliang Nong, Xiaozhu Long, Yuchun Lu

**Affiliations:** ^1^ College of Electronic Information, Guangxi Minzu University, Nanning, China; ^2^ Guangxi Key Laboratory of Intelligent Unmanned System and Intelligent Equipment, Nanning, Guangxi, China; ^3^ Guangxi Key Laboratory of Hybrid Computation and IC Design Analysis, Nanning, Guangxi, China; ^4^ State Key Laboratory for Conservation and Utilization of Subtropical Agro-bioresources, Nanning, Guangxi, China; ^5^ Technology Development Center, Guangxi Agricultural Machinery Research Institute, Nanning, China; ^6^ Department of Technical Research and Development, Nanning Titanium Silver Technology Co., Nanning, China

**Keywords:** sugarcane stem node detection, SimAM, deformable convolution, WIoU, YOLOv7

## Abstract

**Introduction:**

Sugarcane stem node detection is one of the key functions of a small intelligent sugarcane harvesting robot, but the accuracy of sugarcane stem node detection is severely degraded in complex field environments when the sugarcane is in the shadow of confusing backgrounds and other objects.

**Methods:**

To address the problem of low accuracy of sugarcane arise node detection in complex environments, this paper proposes an improved sugarcane stem node detection model based on YOLOv7. First, the SimAM (A Simple Parameter-Free Attention Module for Convolutional Neural Networks) attention mechanism is added to solve the problem of feature loss due to the loss of image global context information in the convolution process, which improves the detection accuracy of the model in the case of image blurring; Second, the Deformable convolution Network is used to replace some of the traditional convolution layers in the original YOLOv7. Finally, a new bounding box regression loss function WIoU Loss is introduced to solve the problem of unbalanced sample quality, improve the model robustness and generalization ability, and accelerate the convergence speed of the network.

**Results:**

The experimental results show that the mAP of the improved algorithm model is 94.53% and the F1 value is 92.41, which are 3.43% and 2.21 respectively compared with the YOLOv7 model, and compared with the mAP of the SOTA method which is 94.1%, an improvement of 0.43% is achieved, which effectively improves the detection performance of the target detection model.

**Discussion:**

This study provides a theoretical basis and technical support for the development of a small intelligent sugarcane harvesting robot, and may also provide a reference for the detection of other types of crops in similar environments.

## 1 Introduction

Sugarcane is the main raw material for sugar production. Although China’s sugarcane cultivation area is large, it is mostly planted in hilly areas, which is not conducive to the work of existing large and medium sized sugarcane harvesters (Zeng et al., 2012). Therefore, the research of miniaturized and intelligent sugarcane harvesters is a development trend, and the recognition of sugarcane stem nodes to judge the position of sugarcane nodes cutting is the first step to realizing intelligent sugarcane harvesting operation.

In the study of recognition of sugarcane stem nodes, Moshashai et al. (2008) first investigated the recognition method of sugarcane stem nodes by comparing the diameters of different parts of sugarcane. Lu et al. (2010) proposed a support vector machine-based feature extraction and recognition method for sugarcane stem nodes, and the recognition rate reached 94.118%. Huang et al. (2013) searched the edges of grayscale images by soble operator and achieved 100% recognition rate in detecting and locating sugarcane nodes using random transform. Meng et al. (2019) proposed a sugarcane stem node recognition algorithm based on multi-threshold and multi-scale wavelet transform, applied to stem node recognition of leaf stripped sugarcane with 100% recognition rate. Zhou et al. (2020) proposed a sugarcane stem node recognition based on Sobel edge detection based sugarcane stem node recognition method, the recognition rate of 93% can meet the working requirements of a sugarcane seed-cutting machine. Chen et al. (2021a) proposed a sugarcane node recognition algorithm based on the minimum point local pixel sum of vertical projection function and analyzed the recognition of single and double nodes, where the recognition rate of a single node is 100% and the recognition rate of double nodes is 98.5%. The above methods mainly rely on traditional image processing methods, which need to work in simple environments and cannot meet the requirements of real-time detection in complex backgrounds.

In recent years, with the development of deep learning technology and the continuous open source of classical target detection algorithms such as Faster-RCNN and YOLO series, the algorithms of deep learning have been widely used in the field of agriculture. As a single-phase detection method, the YOLO algorithm has the characteristics of fast speed and high efficiency compared to the two-phase detection method, which is widely used in target detection in real scenes. Zhang et al. (2022) proposed an improved lightweight network based on YOLOv5s to achieve all-weather detection of dragon fruit in complex orchard environments, introduced a ghost module in yolov5 to realize the lightweight of the model, added a coordinate attention mechanism so that the model can accurately locate and identify dense dragon fruit, and adopted the SIoU loss function to improve the convergence speed, and the results show that the average precision (mAP) of the model is 97.4%. Zang et al. (2022) proposed an improved attention mechanism based on YOLOv5s for detecting the number of small-scale wheat spikes and better solving the problem of occlusion and cross-overlapping of wheat spikes. The university’s channel attention module (ECA) is introduced in the C3 module of the YOLOv5 backbone structure. The results show that the improved YOLOv5s model achieves an accuracy of 71.61% in the wheat spike counting task, which is 4.95% higher than the original model, and the method improves the applicability in complex field environments. Wang et al. (2022a) proposed an improved YOLOv4 model for the accurate detection of pear blossoms in natural environments, which consists of the SENet (squeeze - and - excitation Networks) module-embedded ShuffleNetv2 replaces the original backbone network of the YOLOv4 model and constitutes the backbone network of the YOLO-PEFL model. The experimental results show that the YOLO-PEFL model has an average accuracy of 96.71% and can accurately detect pear blossoms in the natural environment. Li et al. (2019) established an intelligent recognition convolutional neural network model by improving the YOLOv3 network, and the recognition accuracy of stem nodes was 96.89%, however, sugarcane samples were preprocessed by manually removing leaves in a preprocessed monochromatic background environment. To promote sugarcane precut seeds good seeds and good method planting technology, Wang et al. (2022b) proposed an algorithm to improve YOLOv4-Tiny to achieve accurate and fast identification and cutting of sugarcane stem nodes, and the detection accuracy of the improved algorithm was 97.07%.Zhu et al. (2022) proposed a new method of binocular localization based on improved YOLOv4 for the difficult spatial localization of sugarcane nodes using robots under agricultural conditions, and lightened YOLOv4 for porting to embedded chips by network slimming techniques. The results showed that the improved YOLOv4 algorithm reduced the model size, parameters, and FlOPs by about 89.1%, which greatly reduced the complexity of the model. The complexity of the model is greatly reduced, but the accuracy is also slightly reduced. Chen et al. (2021b) proposed a deep learning-based target detection algorithm for the problem of low accuracy of sugarcane stem node recognition in the natural environment and improved the robustness and generalization ability of the algorithm by data set expansion method, and the results showed that the average accuracy was 95.17%. Although good accuracy is obtained, the use of the dataset expansion method is likely to lead to data over fitting.

Deep learning-based target detection methods have already achieved good results in sugarcane stem node recognition in simple backgrounds, but in the field, there is the problem of difficulty in recognizing and accurately locating sugarcane stem nodes in complex environments constituted by light, shading, and other characteristics of the crop such as dense sugarcane and different maturity levels. Therefore, a target detection model based on improved YOLOv7 is proposed in this paper, and the main contributions are as follows:

1. Establishing sugarcane datasets in complex environments.
2. In this paper, an improved target detection network model for complex environments based on YOLOv7 is proposed. The Deformable Convolution Network (DCN) is introduced to replace part of the convolutional layer of the feature extraction network in the original YOLOv7 so that the feature extraction network can adaptively extract the positional features such as occlusion and overlap that leads to the lack of information of sugarcane stems and nodes and the SimAM is added to the ELAN module in the backbone network and the concatenation layer in the feature fusion module. An attention mechanism is added to the ELAN module in the backbone network and the Concat connection layer in the feature fusion module to enhance the extraction ability of the model for small and dense sugarcane stem node features without increasing the complexity of the model. The WIoU loss function is used instead of the original loss function to solve the sample quality imbalance problem, which improves the convergence speed of the model during training.
3. The superiority of our model in the task of target detection in complex environments is verified from different perspectives through comparative and ablation experiments, and the experiments provide ideas and rationale for small intelligent sugarcane harvesters to recognize sugarcane stem nodes.

## 2 YOLOV7 network model and improvements

### 2.1 YOLOv7 model

The YOLOv7 (Wang et al., 2023) target detection algorithm, introduced by the original YOLOv4 (Bochkovskiy et al., 2020) research team in July 2022, is a novel and excellent detector that uses instead of efficient aggregation network, the ELAN module that appears in the network structure, to effectively enhance the network learning capability compared to the previous YOLO series. The YOLOv7 network structure mainly consists of the Input layer, Backbone layer, Neck layer and Head layer, Backbone layer, Neck layer, and Head layer. The input layer is the input layer, which scales the input image to a fixed size to meet the input size requirement of Backbone. The backbone layer is the feature extraction layer, and based on YOLOv5, ELAN structure, and MP structure are introduced. Among them, the ELAN structure consists of different convolutional blocks stacked without changing the width-height of the input feature layers and enhances the interaction between each feature layer through expansion, random combination, and splicing to improve the learning ability of the model. The MP structure consists of convolutional blocks of 3x3 size and a Maxpool dual path, which compresses the width-height of the input feature layers to enhance the feature fusion ability of the network. The neck feature fusion network (Neck layer) includes CBS, SPPCSPC, MP, and ELAN, which follow the traditional PAFPN structure to extract three feature layers located in the middle, lower middle, and bottom layers of the backbone part, respectively, to achieve full fusion of multi-scale features. the SPPCSPC structure achieves a full fusion of multi-scale features by introducing a convolutional spatial pyramid (CSP) in the spatial pyramid pool (SPP) structure) structure to improve the perceptual field of the network, while multiple pooling operations are added in parallel in a string of convolutions, using residual edges for optimization and feature extraction. The Head layer uses the anchor mechanism to output feature maps at three scales, large and small, and uses the reparameterized structure RepConv to articulate the regular convolution to adjust the number of channels and prediction, and then the final prediction results are obtained through the processing of CIou loss function and nonlinear maxima suppression (NMS).

**Figure 1:**
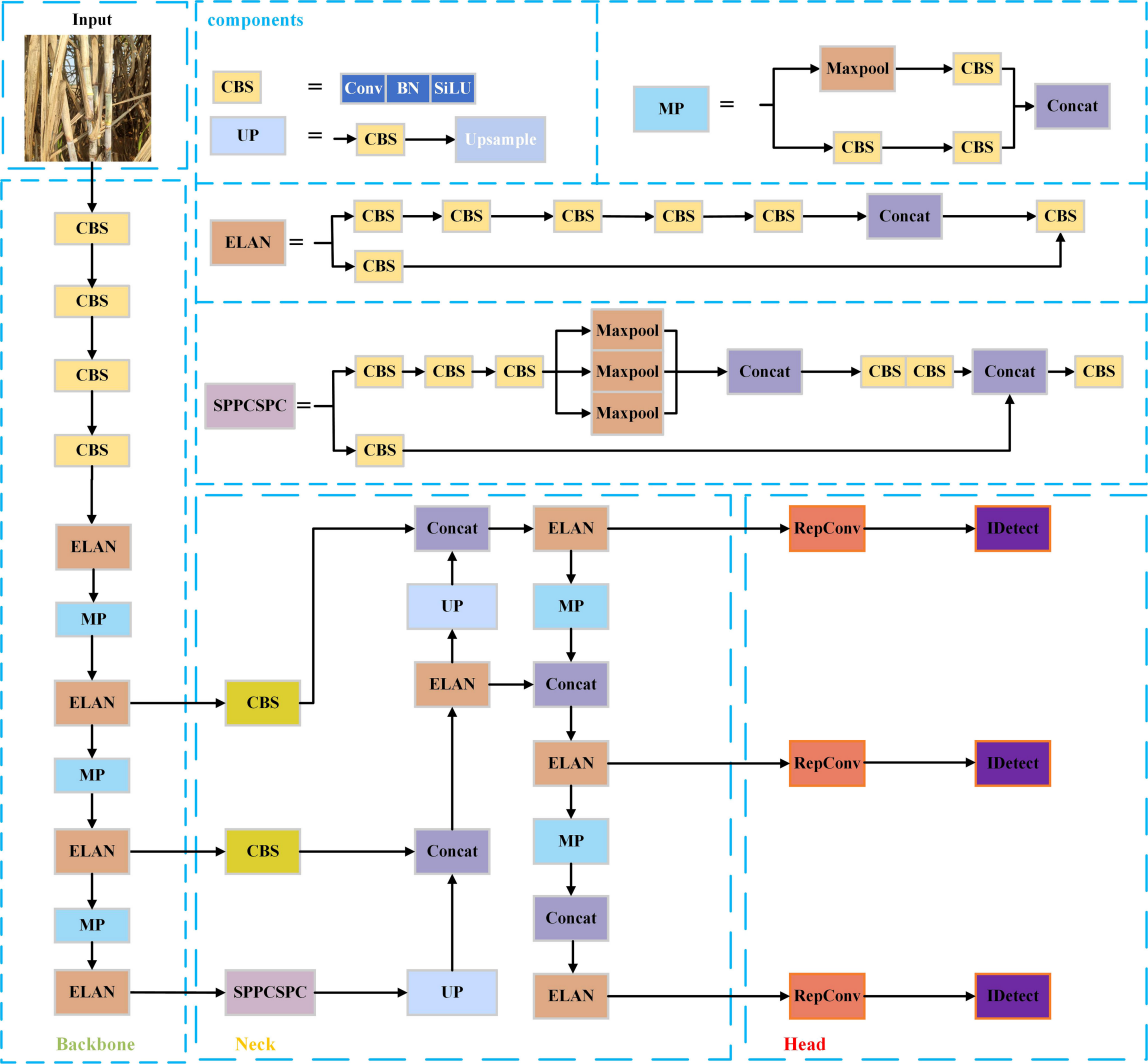
YOLOv7 network structure diagram.

Although the YOLOv7 algorithm performs well in common task scenarios (e.g. pedestrian and vehicle detection), there are still many problems in applying it directly to sugarcane stem node recognition in complex environments: for example, in the actual sugarcane environment, dense clusters of sugarcane, small stem node size, and a large number, and a certain degree of occlusion or overlap will lead to serious cases of missed and false detection. To address the above problems, this paper improves the YOLOv7 algorithm in terms of attention mechanism, convolution layer, and loss function to improve the recognition effect in complex environments. The YOLOv7 network structure is shown in **Figure 1**.

### 2.2 SimAM attention mechanism

**Figure 2:**
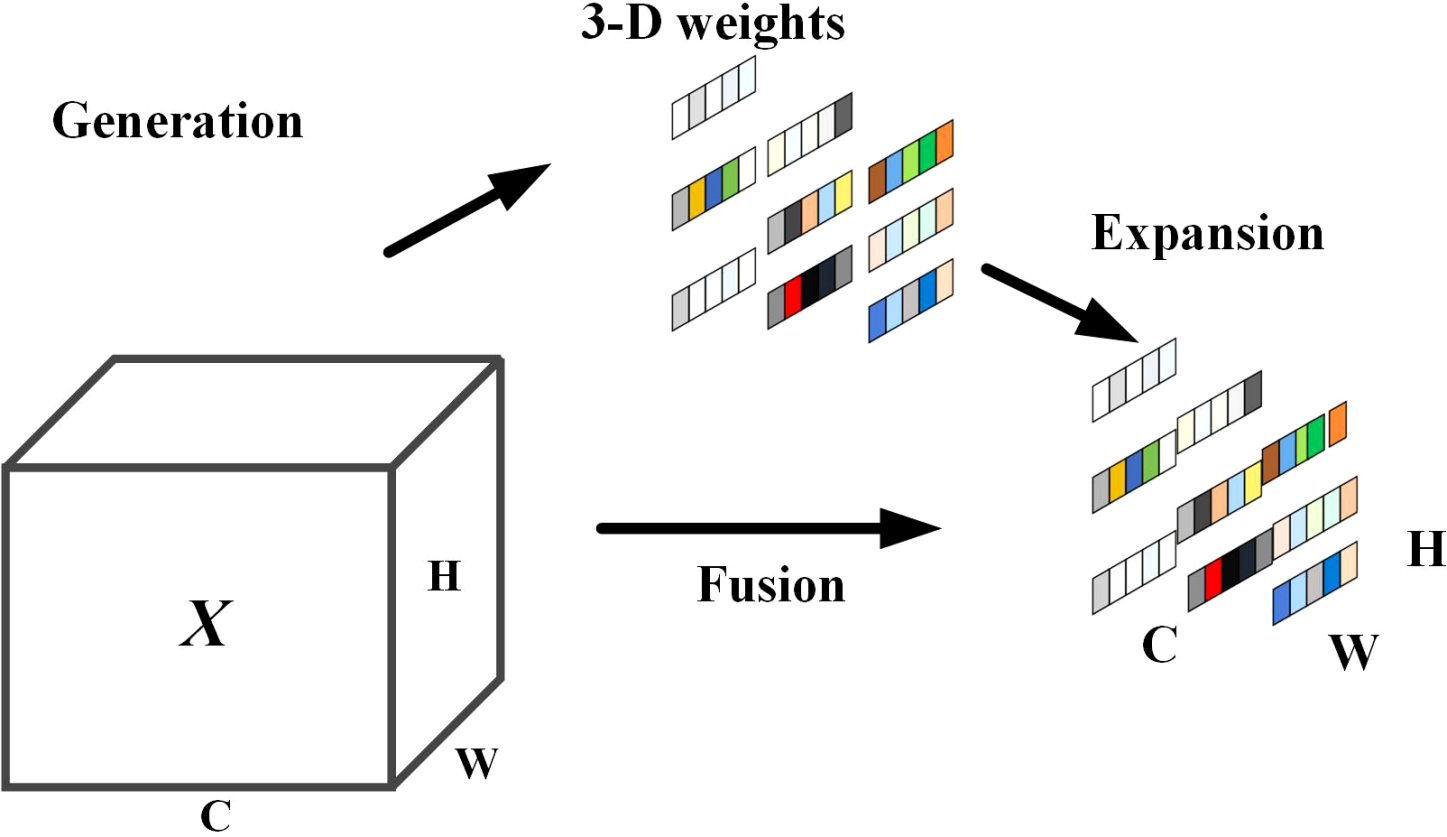
SimAM attention mechanism.

The attention mechanism refers to the model ignoring irrelevant information and focusing on important information by assigning different weights to the input parts of the network, which can effectively improve the feature extraction ability of the model in complex backgrounds. pyramid feature extraction is used as the backbone network in YOLOv7, and in response to the characteristics of dense sugarcane, small target, large number, and easy to obscure and overlap in the natural environment, this paper adds an attention mechanism to the backbone In this paper, we add an attention mechanism to the backbone network to improve the feature extraction capability and enhance the feature representation capability. The traditional attention mechanisms SE (Hu et al., 2018) (Squeeze-and-Excitation), (Woo et al., 2018) CBAM (Convolutional Block Attention Module), ECA (Wang et al., 2020) (Efficient Channel Attention Module), CA (Hou et al., 2021) (Coordinate Attention), all assign attention weights along channels or spatial locations and require additional sub-network structures and model parameters, which on the one hand cannot generate real 3D weights based on channels or spaces, and on the other hand, the additional sub-network structures inevitably lead to an increase in network complexity. In order to solve the shortage of traditional attention mechanisms, this paper proposes to use SimAM (Yang et al., 2021) non-parametric attention mechanism, whose attention weights are assigned as shown in **Figure 2**.

The SimAM attention mechanism generates truly effective 3D weights directly by designing an energy function, without adding additional sub-networks or additional model parameters. The design of the energy function is inspired by neuroscience theory and aims to measure the linear differentiability between neurons to find the important neurons, and for each neuron of the input, the minimum energy function is defined as follows.


(1) $$e_{t}^{*}=\frac{4\;({\hat{\sigma }}^{2}+\lambda )}{{\left(t-\hat{\mu }\right)}^{2}+2{\hat{\sigma }}^{2}+2\lambda }$$

where *t* denotes the target neuron of the input feature in the current channel, where *µ*,*σ* ^2^, is the mean and variance of all neurons in the channel to avoid repeated calculations and reduce the computational cost, and *λ* is the weight constant. To better realize attention, the SimAM module needs to assess the importance of each neuron. In neuroscience, neurons with rich information usually exhibit different firing patterns than surrounding neurons. In addition, activated neurons tend to inhibit surrounding neurons, spatial inhibition, and neurons with spatial inhibition should be given higher importance (Webb et al., 2005). Equation (1) reveals a phenomenon: the lower the energy t of a neuron, the more it differs from surrounding neurons, and therefore the more significant its contribution to visual processing. Thus, the importance of each neuron can be evaluated in terms of $e_{t}^{*}$ . SimAM attention mechanism is added to the ELAN module of the backbone network as well as the Concat connection layer of the neck network, SimAM adjusts the distribution of the attention of the feature map by evaluating the importance of each neuron, so that the channel and the spatial attention work in synergy, the importance of the neurons of the sugarcane stem node will be calculated through the training model, and the information of the secondary disturbances other than the sugarcane stem node will be suppressed, so that the information of the secondary disturbances other than the sugarcane stem node will be suppressed, thus weaken the influence of complex environmental factors on sugarcane stem node recognition. At the same time, SimAM can adaptively adjust the weights of feature mapping and pay more attention to the local area of the target. This can improve the target localization accuracy, reduce the localization error and enhance the feature extraction ability of the backbone network.

The output equation of the attention module is:


(2) $$Y=sigmoid\left(\frac{1}{E(X)}\right)☉X$$

The final output is obtained by adding the Sigmoid function to suppress the outliers of the attention weights and performing the dot product operation with the corresponding elements of the input feature matrix.

Aiming at the recognition of sugarcane stem nodes in complex environments and the lack of an attention mechanism in the YOLOv7 network, this paper effectively extracts finer-grained feature information by adding the SimAM attention mechanism to the last layer of the 1×1 CBS of the ELAN module in the Backbone module of YOLOv7 and by incorporating the Concat in the Neck into the SimAM attention mechanism.

### 2.3 Deformable convolution

**Figure 3:**
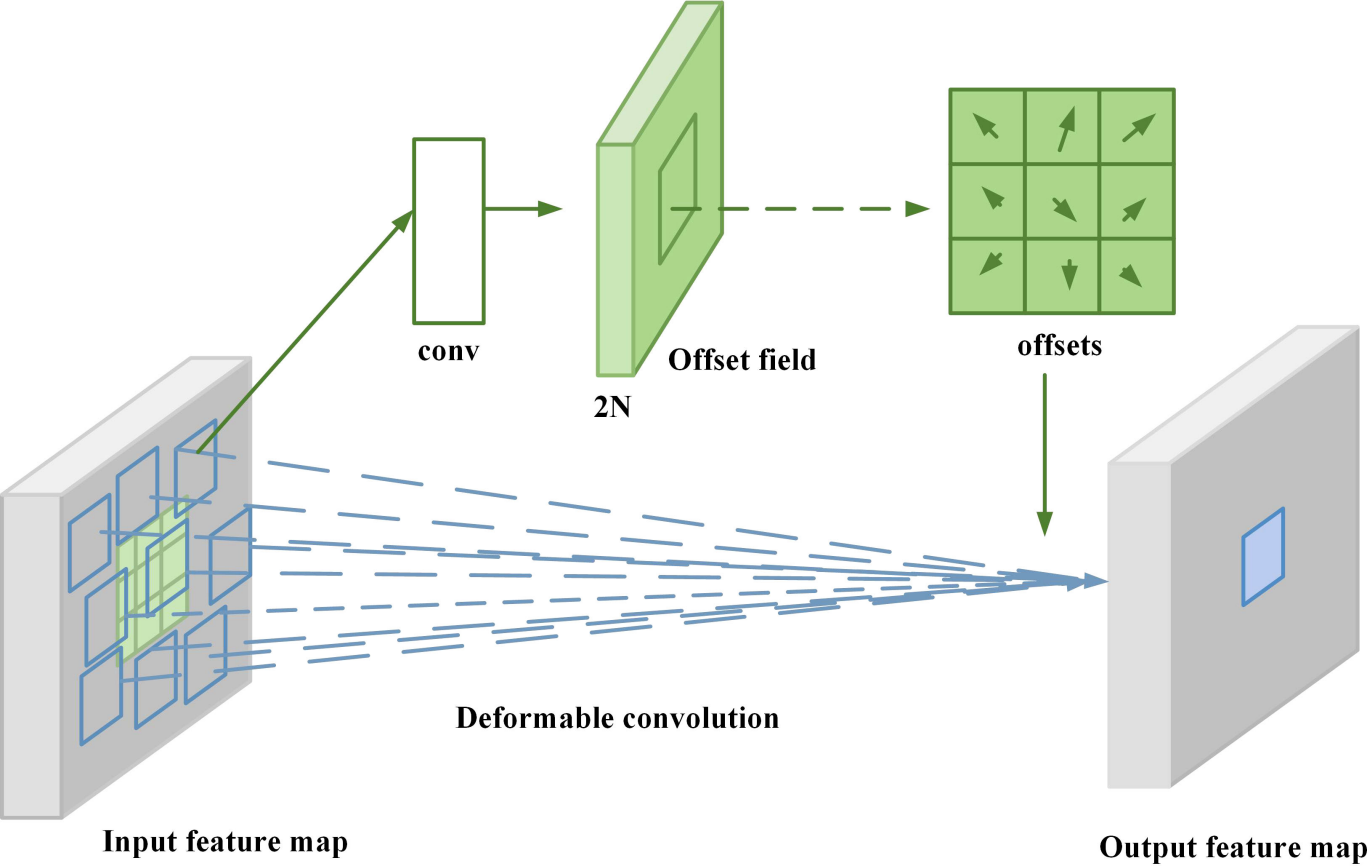
Deformable convolutional network diagram.

In the original YOLOv7 model, ordinary convolutional blocks are used in the feature extraction network Backbone, which mainly consists of a traditional convolutional layer, BN layer, and activation function, and due to the fixed size of the convolutional kernel of the traditional convolutional layer, it has poor robustness to unknown geometric transformations and poor generalization ability. When performing feature extraction, it is difficult for the fixed-size convolution kernel to extract the boundary information of the object accurately because the size and contour of different objects are generally different, which affects the ability of the network to extract the features of the object. The traditional convolutional calculation method is as follows:


(3) $$y\left(p_{0}\right)=\sum_{p_{n}\in R}W\left(p_{n}\right)\cdot X\left(p_{0}+p_{n}\right)$$

where *R* denotes the size of the partial feature map corresponding to the convolution kernel, *W* (*p_n_*) denotes the weight corresponding to the nth sampled point in the sampled region, and *X* (*p*_0_ + *p_n_*) denotes the pixel value size of the nth sampled point. To solve the above problem, a deformable convolutional network (Dai et al., 2017) is introduced in this paper, as shown in **Figure 3**:

The network is able to adaptively adjust the size and shape of the convolution kernel for objects of different shapes, which has better robustness and generalization ability compared with traditional convolutional networks, thus enhancing the underlying network’s ability to extract object features. In the variable convolution operator, adding a learnable offset parameter offset to each element in the convolution kernel can make the originally fixed convolution kernel have the ability to adapt to the object shape, and the computational equation is as follows:


(4) $$y\left(p_{0}\right)=\sum_{n\in R}W\left(p_{n}\right)\cdot X\left(p_{0}+p_{n}+\Delta p_{n}\right)$$

In the variable convolution calculation equation (3), Δ*p_n_* denotes the offset. Considering the irregularity of the sampling position of the variable convolution, so that the offset is generally fractional, equation (4) is implemented with bilinear interpolation as follows:


(5) $$y\left(p\right)=\sum_{q}G\left(q,p\right)\cdot X\left(q\right)$$

where *p* denotes an arbitrary position in the region corresponding to the deformable convolution, *q* is the pixel value corresponding to a sampling point in the feature map *X*, and *G*(*q,p*) denotes a two-dimensional bilinear interpolation kernel. In the sugarcane stem node detection task, sugarcane may have complex deformations, such as cane twisting, deformation, or attitude changes, which have an impact on the accurate identification of sugarcane stem nodes. Traditional fixed convolution kernels are difficult to capture these local deformations. Deformable convolution adjusts the sampling position of the convolution kernel by introducing offsets, which allows the convolution operation to better adapt to the target’s deformations and enhances the model’s ability to recognize complex shapes. Deformable convolution utilizes additional convolutional layers to learn the corresponding offsets, and superimposes the obtained offsets on the corresponding pixels in the input feature maps, allowing the convolutional kernel to diverge the sampling in the input feature maps, so that the network can focus on the target function. The embedded deformable convolutional layer can adaptively adjust the sensory field size and position during the convolution process, so that the sampling position around each position during pooling is adaptive and better adapts to the shape and size of the sugarcane stem nodes, thus improving the detection accuracy.

**Figure 4:**
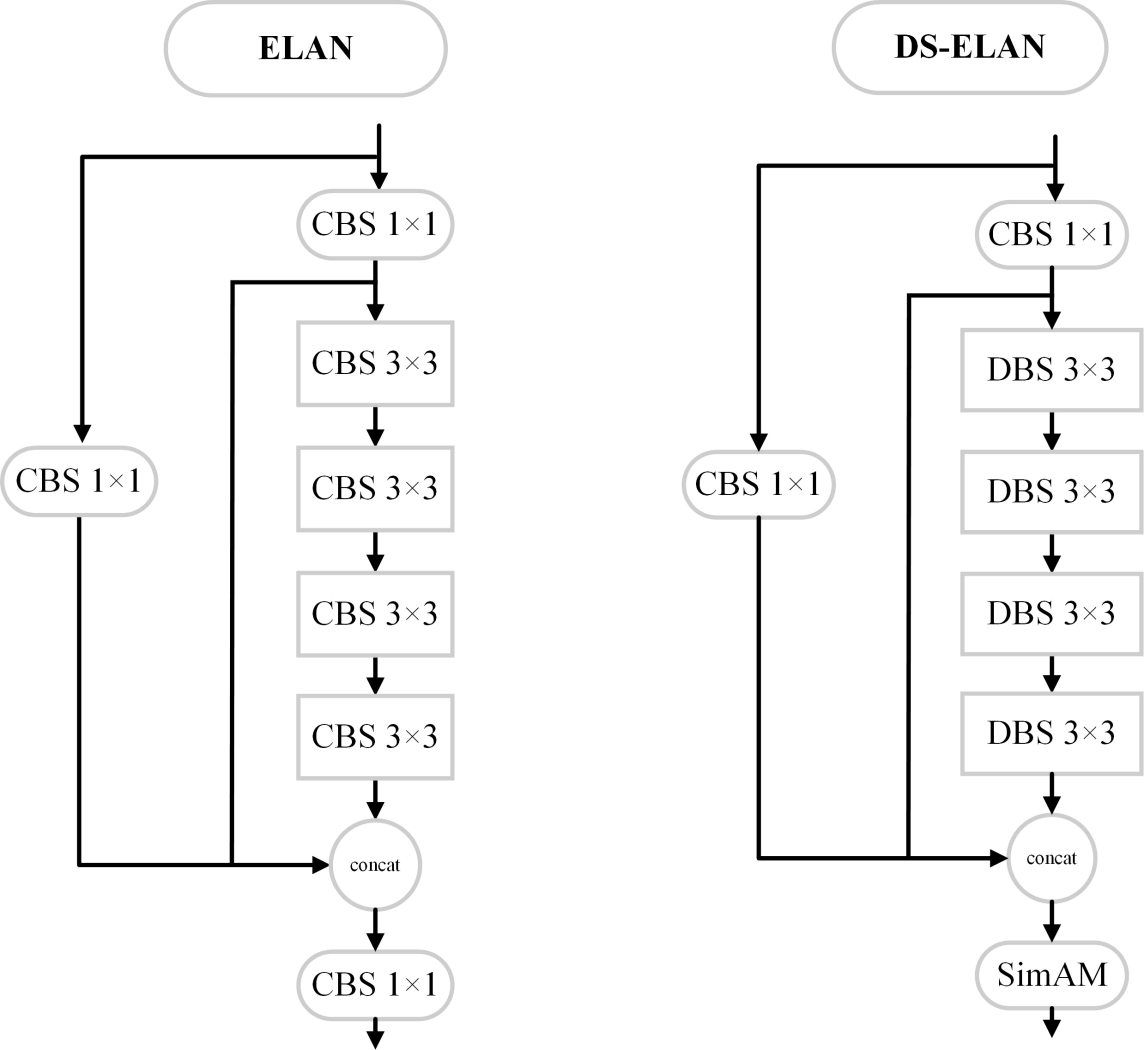
DS-ELAN structure diagram.

Since deformable convolution adds one more parameter compared to conventional convolution, the 3 × 3 convolution kernel in the ElAN module of the backbone network is replaced with deformable convolution (DCN) in the improved algorithm of this paper, and DCN, BN, and SiLU form the DBS module. And the last layer of 1 × 1 CBS in ELAN is replaced with the SimAM attention mechanism, as shown in **Figure 4**, and the replaced structure is represented by DS-ELAN, which enhances the network with a smaller increase in the computational capability of feature extraction and complex background target detection.

### 2.4 Loss function improvement

The YOLOv7 network uses CIoU (Zheng et al., 2021) Loss as the Bounding Box loss function of the model. This function is mainly used for the regression of the prediction box so that the prediction box of the object is closer to the position of the object labeled bounding box. In the model training, CIoU Loss calculates the distance between the prediction box and the center of the real bounding box, the overlap area, and the aspect ratio of the two boxes for the regression of the bounding box, but it does not take into account the balance of the quality of the training samples, which will lead to the slow convergence and low efficiency of the network, and may result in a worse model due to the random matching of the prediction boxes during training. CIoU is calculated as follows:


(6) $$L_{CloU}=1-I_{IoU}+\frac{\rho^{2}(b,\;b_{gt})}{c}+\alpha$$

(7) $$v=\frac{4}{\pi^{2}}{\left(\arctan\frac{w_{gt}}{h_{gt}}-\arctan\frac{w}{h}\right)}^{2}$$

(8) $$\alpha =\frac{v}{(1-I_{IoU})+v}$$

Where: *b* denotes the centroid of the prediction frame; *b*_gt_ denotes the centroid of the true frame; *ρ* represents the Euclidean distance between the two centroids is calculated, and *c* denotes the diagonal length of the minimum enclosing frame covering the prediction frame and the true frame;*α* is the balance parameter; *v* is used to measure whether the aspect ratio is consistent. It can be seen from Equation (7) that when the aspect ratio of the prediction frame and the true value are equal and v takes 0, the penalty term for the aspect ratio in the loss function in CIoU degenerates to 0, resulting in a penalty failure and the final prediction frame cannot fit the true frame. When the prediction frame fits the target frame well, a good loss function should be able to attenuate the penalty of geometric factors.

Because the training data inevitably contains low-quality examples, geometric measures such as distance and aspect ratio can exacerbate the penalty on low-quality examples thus degrading the generalization performance of the model.Wise Iou (Tong et al., 2023) can well improve the sample quality imbalance problem and increase the accuracy of the target detection algorithm. The WIoU loss function is formulated as follows:


(9) $$L_{WIoUv1}\;=\;R_{WIoU}L_{IoU}$$

(10) $$R_{WIoU}=\exp\left(\frac{{\left(x-x_{gt}\right)}^{2}+{\left(y-y_{gt}\right)}^{2}}{{\left(W_{g}^{2}+H_{g}^{2}\right)}^{*}}\right)$$

*R_WIoU_* range is [1,e), which significantly amplifies the *L_IoU_* of the common quality anchor box.*L_IoU_* range is [0,1]. will significantly reduce the *L_IoU_* of the high-quality anchor frame and the distance between its center of attention when the anchor frame overlaps well with the target frame.

where *x y* is the coordinate of the center point of the prediction frame, *x*_gt,_ *y*_gt_ is the coordinate of the center point of the real frame, and *Wg*, *Hg* denote the width and height of the minimum enclosing frame. In order to prevent the generation of gradients that hinder convergence, *Wg* and *Hg* are separated from the computational graph (the superscript * indicates this operation), which effectively eliminates the factors that hinder convergence, so no new metric, such as aspect ratio, is introduced. Since L*_IoU_* is dynamic, the quality classification criteria of the anchor boxes are also dynamic, which allows WIoU to make a gradient gain allocation strategy that best fits the current situation at each moment.

This strategy reduces the competitiveness of high-quality anchor boxes and also reduces the deleterious gradients generated by low-quality samples. In the sugarcane stem node detection task, some samples may be challenging due to the diversity and complexity of sugarcane images, such as blurred, occluded, or small size of sugarcane images. For these low-quality examples, they may generate noisy or unreliable gradient signals that interfere with the model training process. By introducing category weights through multiple, the WIoU loss function can reduce the weights of low-quality samples relatively, which reduces the impact of these samples on the model parameter updates. Therefore, by reducing the competitiveness of high-quality anchor boxes and reducing the harmful gradient of low-quality samples, the WIoU loss function is able to focus more on the optimization of average-quality anchor boxes and improve the performance of the target detector in general. This strategy helps to make the model more focused on the detection accuracy of important target categories and enhances its ability to handle medium-quality samples, thus improving the effectiveness and performance of overall target detection.

Therefore, this paper uses the WIoU loss function to replace the CIoU in the original network to solve the problem that the regression boxes cannot be matched accurately due to the unbalanced sample quality.

**Figure 5:**
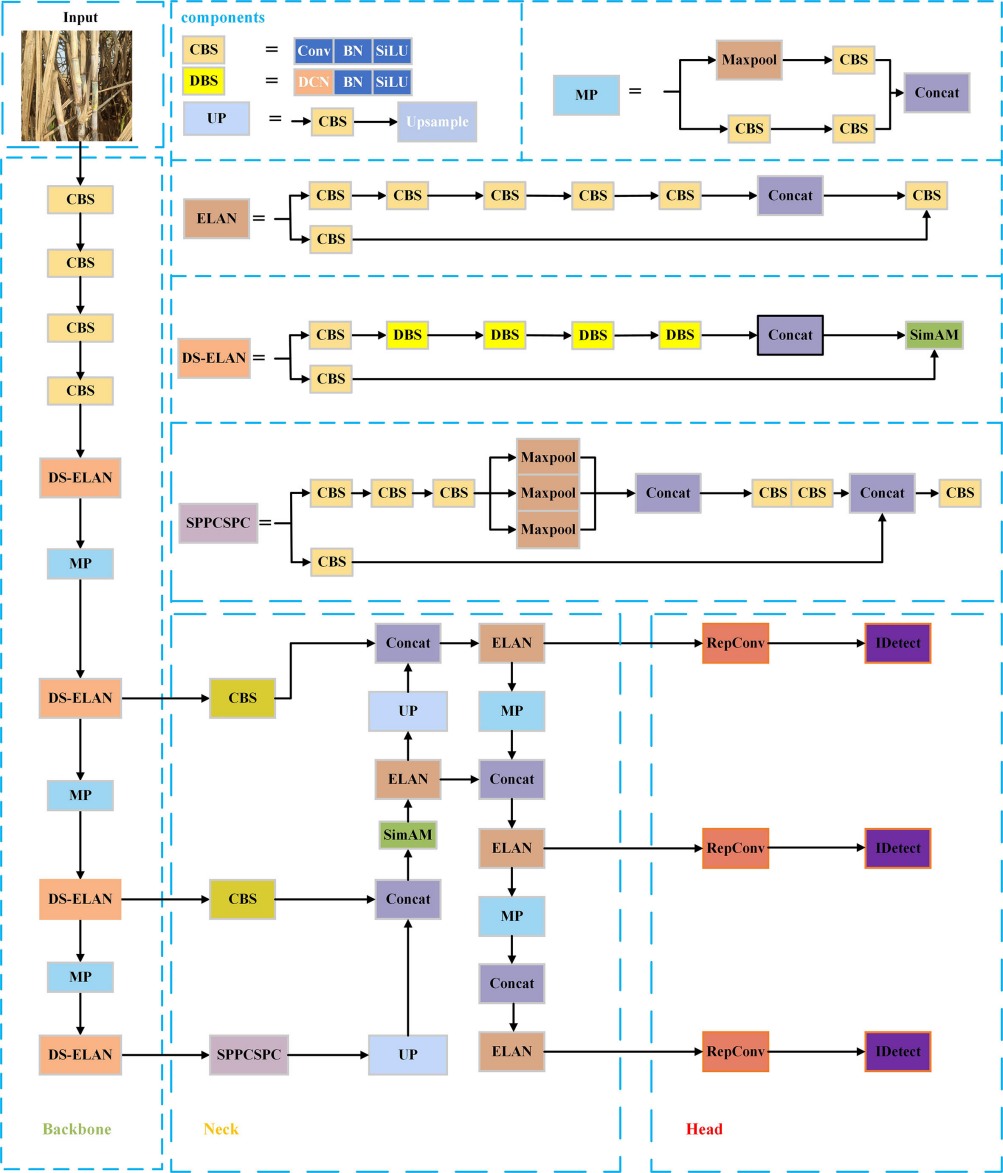
Improved YOLOv7 network structure diagram.

In summary, the improved algorithm adds the SimAM attention mechanism to the feature fusion part of the original YOLOv7 backbone network to enhance the network’s ability to extract target feature information in complex scenes. By using deformable convolution to replace some ordinary convolution layers, the convolution kernel is deformed by more pairs of convolution kernels, which in turn enhances the perceptual field and feature extraction ability of the convolutional network, so that it can better adapt to the shape and position changes of target objects, thus improving the accuracy of object recognition and localization. The CIoU is replaced by WIoU to solve the sample mass balance problem and to make the prediction frame fit the real frame better to improve the detection accuracy. The structure diagram of the improved YOLOv7 network is shown in **Figure 5**.

## 3 Experiment and analysis

### 3.1 Experimental data set

**Figure 6:**
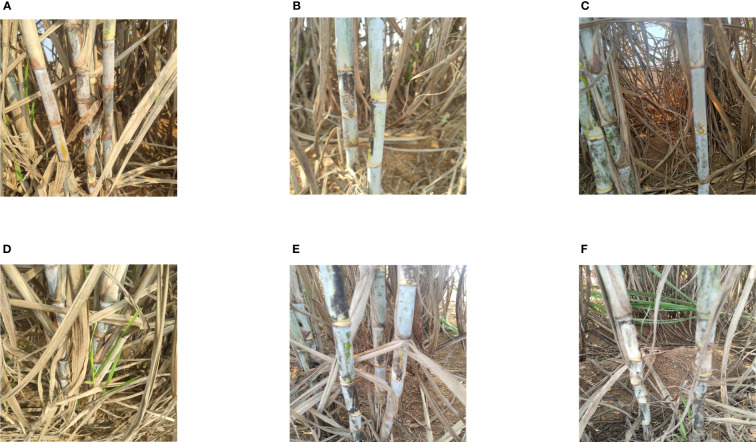
Partial data set display: **(A)** Sunbeam; **(B)** Back lighting; **(C)** Light blocking; **(D)** Leaf wrap; **(E)** Foliage shade; **(F)** Weed shading.

**Figure 7:**
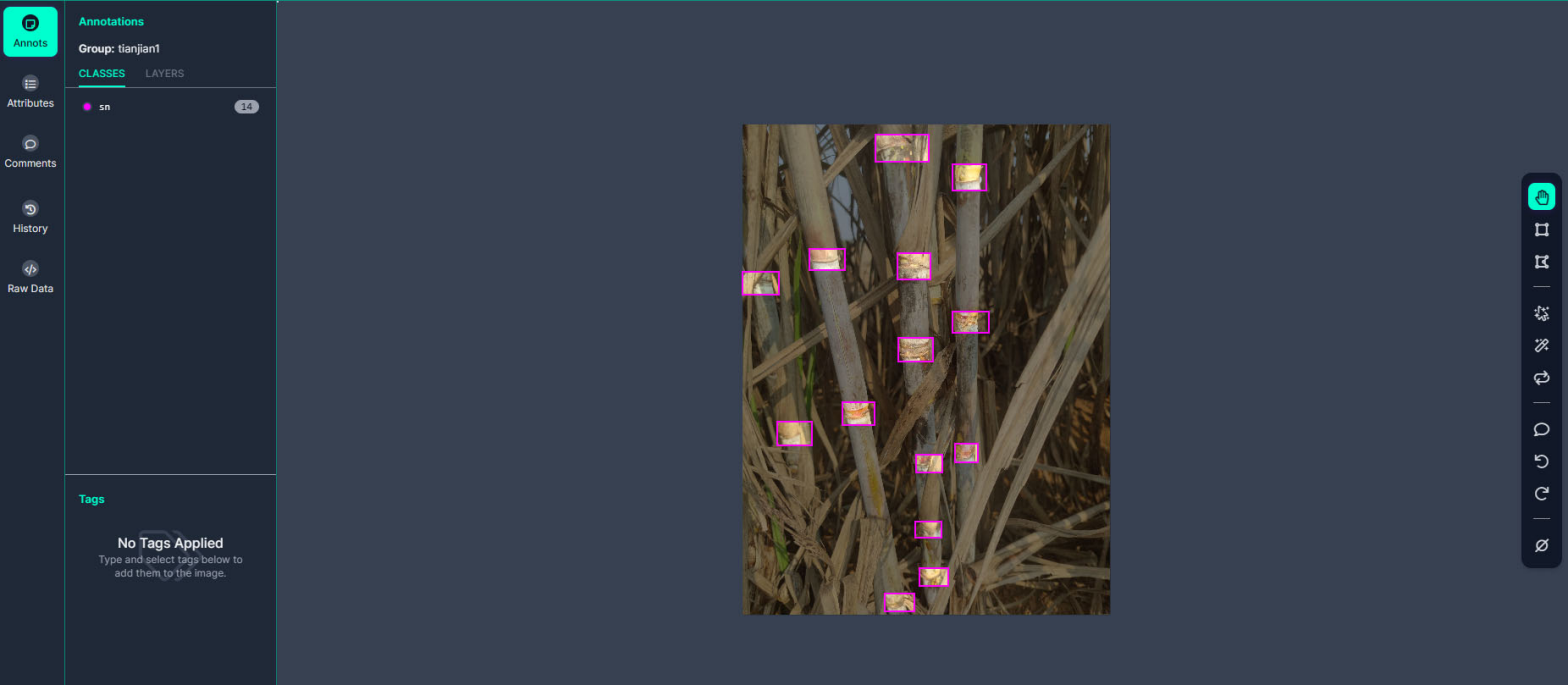
Marking process.

The sugarcane images used in this paper were taken from Su Village, Tao Wei Town, Hengxian County, Guangxi, China, which is a sugarcane plantation. The images were captured by a Xiaomi 10pro digital camera with a resolution of 1080 × 1440 pixels, and the shooting time periods were morning, noon, and afternoon, and a total of 2144 different sugarcane images were obtained by constantly changing the distance and shooting angle. They contain images under uneven conditions of natural scenes such as leaf occlusion, overlapping occlusion, visual similarity to the background image, dense target, backlight, front light, and side light, and are saved in JPG format. The following **Figure 6** shows some images taken under different conditions, which were randomly divided into 80% as the training set, 10% as the validation set, and 10% as the test set, forming 1715, 214, and 214 images for model training and testing, respectively. The datasets were then annotated, and the sugarcane stem node bounding boxes in each image were drawn manually using the Roboflow annotation platform, and the annotation files were saved in YOLO text format, which requires the target class, coordinates, height, and width. The process of annotating the sugarcane stem nodes is shown in **Figure 7**.

### 3.2 Model evaluation metrics

This paper adopted evaluation metrics including precision (*P*), recall (*R*), mean average precision (*mAP*), and F1 score.

*P* and *R* refer to the precision and recall of the detection model, respectively. Precision represents the proportion of true positive samples in the samples predicted as positive by the classifier. The recall represents the proportion of true positive samples that are correctly predicted as positive by the classifier among all true positive samples. The formula for calculating precision and recall is:


(11) $$P=\frac{TP}{TP+FP}\times 100\%$$

(12) $$R=\frac{TP}{TP+FN}\times 100\%$$

The *F*1 score considers both precision and recall, and it can reflect the stability of a model. A higher *F*1 score indicates a more stable model. The formula for calculating the *F*1 score is:


(13) $$F1=\frac{P\times R\times 2}{P+R}$$

*mAP* is the average precision of each class and the average value of *AP*, its calculation formula is:


(14) $$mAP=\frac{1}{C}\int_{0}^{1}P(R)dR$$

### 3.3 Experimental environment and parameter settings

The experimental environment is a 64-bit Ubuntu 22.04 system with an Intel(R) Xeon(R) Platinum 8157 CPU @ 2.30GHz and an NVIDIA GeForce RTX3090 graphics card with 24GB video memory. The study is based on the PyTorch deep learning framework, and the development environment is PyTorch 1.11.0, Cuda 11.3, and Python interpreter version 3.9. The parameters of the experiments conducted in this experiment are shown in **Table 1**:

**Table 1:** Experimental parameters.

Parameters	Values
Learn rate	0.01
Epochs	100
Batch-size	16
Wokers	4
Img size	640×640
Nms	0.3
Conf thres	0.25

### 3.4 Ablation experiments

To validate the effectiveness of the improvement points in this paper, five sets of ablation experiments were conducted on the sugarcane dataset using the original YOLOv7 network as a baseline and keeping the environment and parameters uniform. The validation criteria include mAP values and F1 values. The experimental results are shown in **Table 2**, where bold font indicates the optimal results in each column and √ indicates the use of the corresponding method.

**Table 2:** Ablation experiments.

Number	SimAM	DCN	WIoU	mAP(%)	F1
No.1				91.10	90.20
No.2	√			92.50	91.13
No.3		√		92.30	91.08
No.4			√	92.23	90.87
No.5	√	√	√	94.53	92.41

(1) No.1 shows the experimental results of the pre-improved YOLOv7 algorithm, which serves as a comparative benchmark for the experiments of the latter 4 groups, detecting an mAP value of 91.10% and an F1 value of 90.20.

(2) No.2 is to add only the attention mechanism, although the attention mechanism increases the amount of computation and the number of parameters, the mAP value is improved by 1.40%, and the F1 value is improved by 0.93.

(3) No.3 is to replace only some of the convolutional layers in the backbone network with deformable convolution, the mAP value is improved by 1.20%, and the F1 value is improved by 0.88.

(4) No.4 for replacing only the WIoU loss function, without increasing the number of model parameters and computational effort, the mAP value is improved by 1.13%, and the F1 value is improved by 0.67.

(5) No.5 shows the experiment of the improved algorithm in this paper, compared with the YOLOv7 algorithm before improvement, the mAP value is improved by 3.43%, F1 value is improved by 2.21, which confirms the validity of each improvement point. "√*" indicates that this methodology is used.

### 3.5 Comparison of experimental results and analysis

In order to evaluate the performance of the algorithms, the improved algorithm proposed in this paper is compared with the algorithms such as YOLOv7, YOLOv5, YOLOv8, Faster-R-CNN, and SSD for the detection performance on the dataset, and all the experiments are conducted under the same parameters. The experimental results are shown in **Table 3**, the table below, the improved algorithm, the mAP reached 94.53% and the F1 score reached 92.41, which are 3.43% and 2.21 respectively higher than the baseline YOLOv7, compared to the other algorithms in **Table 3** with the best overall results. Faster R-CNN and SSD algorithms are affected by the fixed parameters of the anchor frame, which reduces the model detection effect. The YOLOv5 algorithm optimizes the network structure better, and the predicted position regression is more accurate. YOLOv8 has higher accuracy and recall because the C3 structure of the backbone network is replaced by the C2f structure with a richer gradient flow, and the number of channels is adjusted differently for different scale models, which significantly improves the model performance, but it is higher than There is still a certain gap compared with this paper. Comprehensive measurement of different detection algorithms, the improved algorithm in this paper is better, which confirms the effectiveness of the improved algorithm in this paper.

**Table 3:** Comparison experiments.

Models	mAP(%)	F1
YOLOv7	91.10	90.20
YOLOv5	92.34	91.20
YOLOv8	91.41	91.00
Faster-R-CNN	76.89	76.00
SSD	72.48	72.00
Improved-YOLOv7	94.53	92.41

## 4 Discussion

**Figure 8:**
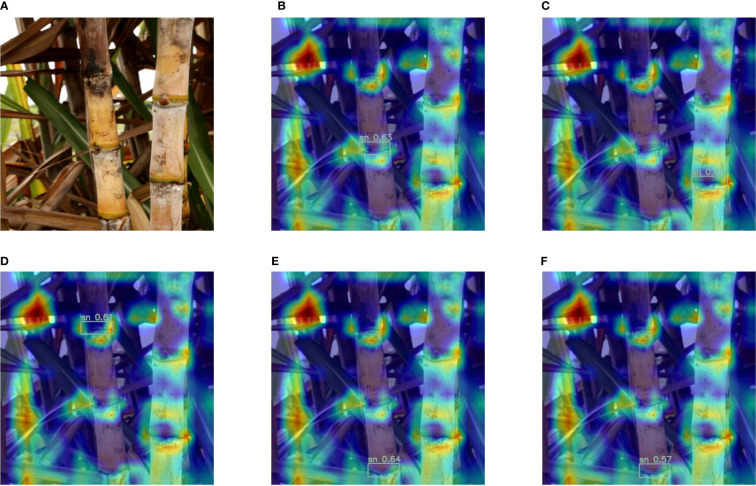
Heat map before adding the attention mechanism: **(A)** Original image; **(B-F)** Heat map of each sugarcane stem node of the original model.

**Figure 9:**
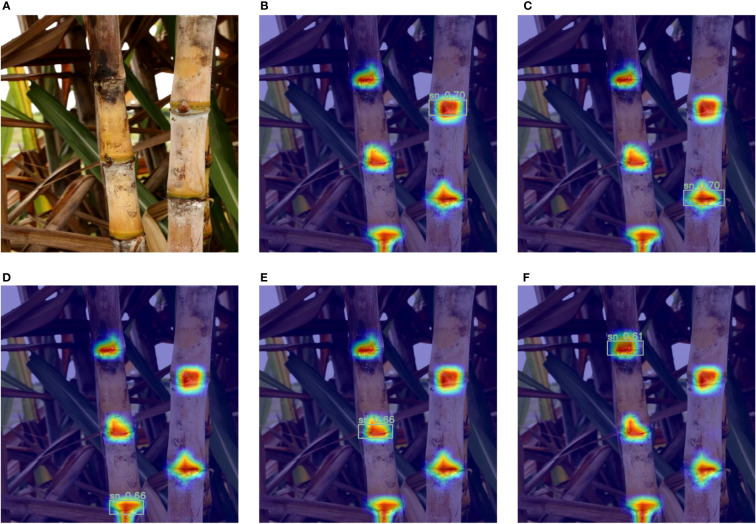
Heat map after adding the attention mechanism: **(A)** Original image; **(B-F)** Heat map of each sugarcane stem node for the improved model.

Below we discuss the visualization results after the introduction of the attention mechanism. In **Figures 8**, **9**, A is the original image of Cane, (B-F) are the heat maps before and after adding the attention mechanism, respectively, and the darker red color in the heat map indicates the larger value. From **Figure 8**, it can be seen that the model is not accurate enough in predicting the stem nodes during the target detection, the darkest colored part is not the sugarcane stem node, and there is a false detection. From **Figure 9**, it can be seen that after adding the attention mechanism, the model is more accurate in locating the stem nodes.

**Figure 10:**
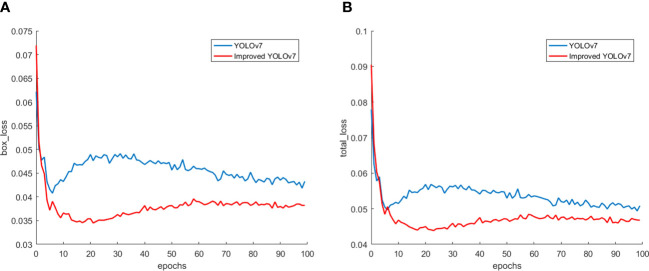
Loss. **(A)** Box loss; **(B)** Total loss.

Here we discuss the effect of the improved loss function. **Figures 10**, show the box loss and total loss before and after the improved algorithm, respectively, and the horizontal coordinates are the number of training rounds. It can be directly concluded from the above figures that the improved algorithm has smaller loss values than the original algorithm, and the values of its box loss and total loss are stable at 0.035, 0.045, and From the figure, we can see that although the improved algorithm has a higher loss value than the original YOLOv7 at the beginning of training, the loss value decreases quickly and stabilizes as the number of training rounds increases. Therefore, it shows that the improved algorithm converges faster and has better performance.

**Figure 11:**
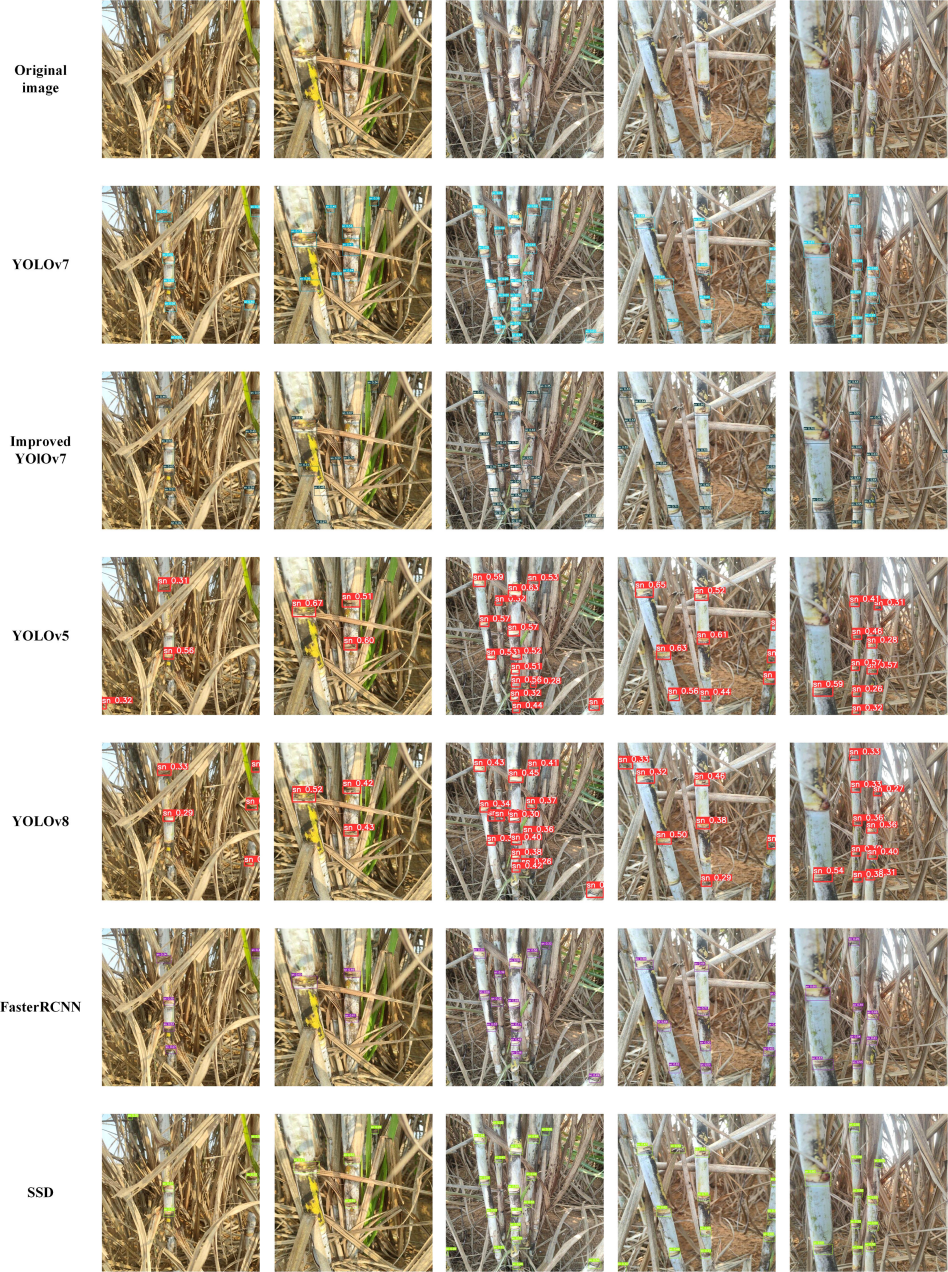
Comparison experiments.

In order to compare the detection effect of the algorithm improvement more intuitively, the detection effect of the original YOLOv7 algorithm, other models, and the improved model of this paper is compared with the real labeled frame as the benchmark, and the detection results of the sugarcane stem nodes are shown in **Figure 11**. It can be seen that in the case of complex background interference, YOLOv7 and other models will have problems such as incomplete detection and misdetection, etc. In this paper, by improving the YOLOv7 model and strengthening the spatial feature extraction ability of the backbone network, the probability of misdetection of the sugarcane stem nodes is significantly reduced. In the case of sugarcane stem nodes with fuzzy edges, unclear contours, and obscured by leaf wrappings, the original YOLOv7 algorithm is prone to miss detection, and the accuracy of sugarcane stem node detection is low. In contrast, the algorithm in this paper, by introducing deformable convolution in the feature fusion network, acquires a larger sensory field and captures more spatial information, and incorporates the attention mechanism in the stem network, which utilizes the multidimensional interaction between channels and space to retain the key information, so that the model can have the ability to differentiate between overlapping targets, and improve the occurrence of leakage detection well. In this paper, the WIoU loss function is introduced to make the model more accurate in judging the position of sugarcane stem nodes and improve detection accuracy. In summary, it shows that the improved model has good applicability in complex environments.

## 5 Conclusion

In this paper, we propose an improved YOLOv7-based sugarcane stem node detection algorithm for dense sugarcane in the natural environment, small target, large number, and easy to obscure and overlap, and verify the effectiveness of the proposed algorithm through experiments. In order to make the feature extraction network adaptive to extract the shape and location features of dense sugarcane stem nodes in the natural environment, a deformable convolutional network is introduced in the module of the original YOLOv7 algorithm, which is used to replace some ordinary convolutional layers to improve the feature extraction ability of the algorithm for dense and occluded sugarcane stem nodes. In the backbone network of YOLOv7, the SimAM attention mechanism is added to improve the network’s ability to extract deep and important features. To improve the matching between the prediction frame and the real labeled frame, the WIoU boundary regression loss function is introduced to replace the CIoU loss function in YOLOv7, which can better guide the network learning and improve the accuracy of detection results. The mAP of the improved YOLOv7 algorithm is 94.53%, which is 3.43% higher than the mAP of the YOLOv7 algorithm, with certain robustness and generalization, and 17.64%, 2.19%, 3.12%, compared with the average accuracy of the Faster-RCNN, YOLOv5, YOLOv8, and SSD network models, respectively, 22.05%, providing a new research idea for the intelligent sugarcane harvester to identify stem nodes. The improved YOLOv7 algorithm can improve the detection accuracy of obscured and overlapping sugarcane stem nodes in the natural environment, but the model detection speed decreases while the accuracy is improved; therefore, in future work, it is still necessary to consider how to further optimize the improved algorithm, use a lighter network while ensuring the detection accuracy and improving its generalization ability, to provide new sugarcane stem node recognition in natural environment Detection methods.

## Data availability statement

The original contributions presented in the study are included in the article/supplementary material. Further inquiries can be directed to the corresponding author.

## Author contributions

Conceptualization, CW and HG; Methodology, CW and HG; Software, HG; Validation, HG, JL, and BH; Formal analysis, YH, and KL; Writing-original manuscript preparation, HG; Writing-review and editing, HG and JL; Supervision, HN, XL, YL, QW. all authors have read and agreed to the published version of the manuscript.
